# Engineered *Lactiplantibacillus plantarum* and *Levilactobacillus brevis* utilizing ribonucleoprotein-mediated editing for inactivation of hemolysin gene

**DOI:** 10.1007/s11274-025-04598-y

**Published:** 2025-10-13

**Authors:** Hea Joon Kim, Min Young Kwon, Seongbong Song, Seong Won Cheon, Hyo Jin Kim

**Affiliations:** 1 Graduate School of International Agricultural Technology, Seoul National University, Pyeongchang, 25354 Republic of Korea; 2 Institutes of Green Bio Science and Technology, Seoul National University, Pyeongchang, 25354 Republic of Korea; 3 Research Institute of Food and Biotechnology, SPC Group Co, 1 Gwanak- ro, Gwanak-gu, Seoul, 08826 Republic of Korea

**Keywords:** CRISPR/Cas9, Genome editing, Hemolysin, Lactic acid bacteria, Ribonucleoprotein

## Abstract

*Lactiplantibacillus plantarum* and *Levilactobacillus brevis* are widely used probiotics with significant potential as chassis organisms for probiotic engineering. However, their bioengineering remains underdeveloped compared to that of other probiotic bacteria due to the limited availability of genetic tools. Although CRISPR-Cas systems have shown promise for genome editing in *Lactobacillus* species, strain- or site-specific targeting challenges must be overcome to enhance their broader applicability. This study aimed to develop a novel editing system with reduced dependency on plasmids and antibiotics in *L. plantarum* WCFS1, *L. plantarum* SPC 72 − 1 and *L. brevis* SPC-SNU 70 − 2 using a Cas9-gRNA ribonucleoprotein (RNP) complex. Although the *hlyIII* gene has been annotated as a hemolysin-related gene in several *Lactobacillus* genomes, no functional hemolytic activity has been definitively demonstrated to date. In this study, *hlyIII* was selected as a target to evaluate genome editing efficiency and to assess its potential relevance to strain safety. To construct Δ*hlyIII* strains, the RNP complex targeting *hlyIII* was separately transformed with recombinase RecE/T and double-stranded donor DNA. As a result, Δ*hlyIII* mutants were obtained under optimized electroporation conditions. Sequencing analysis revealed a 50 bp deletion and the introduction of a stop codon in *hlyIII* across all mutant strains. The hemolytic activity test showed a reduction in free hemoglobin levels in the *ΔhlyIII* strains compared to the wild type: 27.0%, 74.3%, and 5.0% in *L. plantarum* WCFS1, *L. plantarum* SPC 72 − 1, and *L. brevis* SPC-SNU 70 − 2, respectively. These results suggest strain-dependent differences in hemolytic activity and indicate that inactivation of *hlyIII* may contribute to reduced hemolysis, although further validation is needed to clarify its functional role. In conclusion, the *hlyIII* gene was successfully edited in *L. plantarum* and *L. brevis* using Cas9-gRNA ribonucleoprotein-mediated editing, demonstrating the feasibility of this genome editing platform for application in probiotic strains.

**Graphical abstract:**

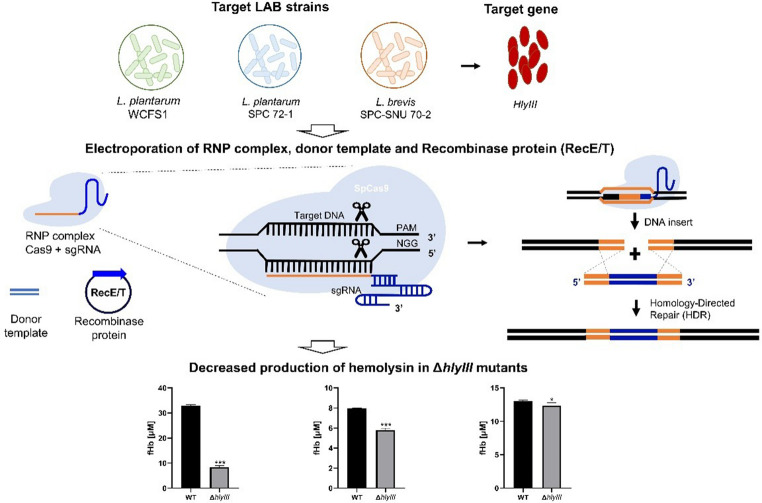

## Introduction

Lactic acid bacteria (LAB), such as *Lactobacillus*, *Lactococcus*, and *Bifidobacterium*, are Gram-positive microorganisms that are extensively utilized in food production because of their ability to ferment carbohydrates into lactic acid (Wang et al. 2021). In addition to their traditional roles, LAB have gained attention for their probiotic properties and metabolic versatility, making them ideal candidates for genetic and probiotic engineering (Mugwanda et al. 2023; Romero-Luna et al. 2022). Probiotic engineering is an emerging discipline that applies genetic and synthetic biological approaches to enhance and optimize probiotic microorganisms. By modifying metabolic pathways, increasing stress resistance, and introducing novel therapeutic functionalities, engineered probiotics can directly produce therapeutic compounds in the host, thereby significantly improving their efficacy and therapeutic potential (Bober et al. 2018; Charbonneau et al. 2020). For example, engineered probiotic strains effectively treat inflammatory bowel diseases, metabolic syndromes, infectious diseases, and even cancer through targeted therapeutic delivery (Al-Fakhrany and Elekhnawy 2024; Liu et al. 2023). Despite these promising developments, genome editing in LAB has encountered significant technical challenges compared to well-established model organisms, such as *Escherichia coli* and *Saccharomyces cerevisiae*. Current genome editing methods depend predominantly on plasmid-based CRISPR-Cas systems, which often exhibit low efficiency, high strain specificity, editing escape, and unintended foreign gene integration (Fanglei and Harold 2021). Recent efforts to improve homologous recombination in LAB, such as the integration of RecE/T recombinase with the CRISPR-Cas system, have successfully demonstrated editing in selected strains, including *L. plantarum*, *L. brevis*, and *Lactococcus lactis* (Guo et al. 2019; Huang et al. 2019; Zhou et al. 2019). However, the broad applicability and scalability of these methods remain limited, necessitating alternative and versatile editing approaches for diverse LAB species (Leenay et al. 2019).

In addition to these technical constraints, the safety concerns associated with probiotics have gained increasing attention. LAB strains, including commonly consumed probiotics such as *Lactiplantibacillus plantarum* and *Levilactobacillus brevis*, have been implicated in bacteremia and sepsis, particularly among immunocompromised individuals (Doron and Snydman 2015; Fanglei et al. 2020; Jeon et al. 2022). Between 2019 and 2021, 48 infections linked to lactobacilli were documented, including three cases involving *L. plantarum* (Rossi et al. 2022). One critical safety factor is the potential for the production of hemolysins, which are toxins capable of damaging red blood cells and posing risks to vulnerable patients (Yasmin et al. 2020). Although no functional hemolytic activity has been definitively demonstrated in LAB to date, hemolysin-encoding genes such as *hlyIII* have been annotated in several LAB genomes, raising questions about their potential roles. This underscores the value of precise genome editing tools for exploring and possibly mitigating strain-specific safety concerns (Chen et al. 2004; Gleb and Nikolai 1996; Oh et al. 2022).

To address technical and safety challenges, our study presents a ribonucleoprotein (RNP)-based genome-editing strategy tailored specifically for LAB. Unlike plasmid-based CRISPR systems, which rely on host transcription and translation to express Cas9 and sgRNA, pre-assembled RNP complexes (comprising the Cas9 protein and sgRNA) enable immediate DNA cleavage by bypassing these cellular processes (Kim et al. 2014; Rubén et al. 2023; Xiao-Hui et al. 2015; Zhang et al. 2021). This method significantly reduces editing time, minimizes off-target effects, and eliminates the introduction of foreign DNA, thereby avoiding potential regulatory and safety questions associated with the long-term persistence of exogenous genetic material. Although RNP-based genome editing has been successfully implemented in eukaryotic models, including plants, animals, and human cells (Arroyo-Olarte et al. 2021; Farboud et al. 2018), its application in Gram-positive bacteria, such as LAB, requires careful optimization owing to challenges in cellular uptake and intracellular stability (Bixi et al. 2021; Zhu et al. 2023).

In this study, we implemented RNP-based genome editing to precisely delete the hemolysin gene (*hlyIII*) in the probiotic strains of *L. plantarum* (WCFS1 and SPC) and *L. brevis* (SPC). The resulting LAB mutants exhibited reduced hemolytic activity to varying degrees across strains, suggesting that *hlyIII* inactivation may contribute to improved safety profiles, although further validation is warranted. Through this approach, we provide a versatile, efficient, and scalable tool for probiotic engineering, significantly advancing the development of safer and more therapeutically potent probiotics.

## Materials and methods

### Strains and primers

All bacterial strains and plasmids used in this study are listed in Table 1. *E. coli* TOP10 (Invitrogen Corporation, Carlsbad, CA, USA) was used as a cloning host. *L. plantarum* SPC 72 − 1 and *L. brevis* SPC-SNU 70 − 2 used in this study were provided by the SPC Research Institute of Food and Biotechnology. *Streptococcus mutans*,* ATCC 6538*, and *Enterococcus faecalis* KCTC 3206 were used as controls for the hemolysis test, as described in previous studies (Baccouri et al. 2019; Wolff and Liljemark 1978; Xu et al. 2024). Plasmids pLH01 (plasmid 117261) and pLH02 (plasmid 117262) were purchased from Addgene (Watertown, MA, USA). Wild-type strains (*L. plantarum* WCFS1, *L. plantarum* SPC 72 − 1, and *L. brevis* SPC-SNU 70 − 2) were cultured at 30 ℃ in deMan Rogosa Sharpe (MRS; Difco, Detroit, MI) medium. *E. coli* TOP10 was grown in Luria-Bertani (LB) medium at 37 ℃. For selection, the antibiotics are supplemented in the medium at the following concentrations: chloramphenicol 12.5 µg/mL for *E. coli*, 10.0 µg/mL chloramphenicol for *L. plantarum* and 6.0 µg/mL for *L. brevis.*

**Table 1:** Primers used in this study

Primers	Sequence (5’ to 3’)	Description
*L.p*_Hem_sgRNA2	**TTTCGTATCTTCGATCACAG**GTTTTAGAGCTAGAAATAGCAAGTTAAAATAAGGCTAGTCCGTTATCAACTTGAAAAAGTGGCACCGAGTCGGTGCTTTT	sgRNA for *HemolysinIII* cleavage
*L.p*_Hem_sgRNA17	**GCGTGTACGTGCTGATTGCT**GTTTTAGAGCTAGAAATAGCAAGTTAAAATAAGGCTAGTCCGTTATCAACTTGAAAAAGTGGCACCGAGTCGGTGCTTTT	
*L.b*_Hem_sgRNA1	**CTTGAAGTAGTAGAATGACG**GTTTTAGAGCTAGAAATAGCAAGTTAAAATAAGGCTAGTCCGTTATCAACTTGAAAAAGTGGCACCGAGTCGGTGCTTTT	
*L.b*_Hem_sgRNA2	**CTACTTCAAGCTCACGCCAA**GTTTTAGAGCTAGAAATAGCAAGTTAAAATAAGGCTAGTCCGTTATCAACTTGAAAAAGTGGCACCGAGTCGGTGCTTTT	
*L.p*_Hem_temp_F	AAGCCGATAATTAGGTTGAATAGGT	Amplification of repair DNA (Double-stranded DNA)
*L.p*_Hem_temp_R	CCAGTTGAAAAACTTTTCGCAATAA	
*L.b*_Hem_temp-F	TATCAAAGGTTTCGACAAACGATCCT	
*L.b*_Hem_temp-R	CTGTTATACGCTTAATCTTAGGGTCAAAT	
*L.p*_Hemolysin_F	GTTCGACGTTATTTCACGGATT	Amplification of hemolysinIII gene
*L.p*_Hemolysin_R	TAAATGACCGTTTCGACCTTTT	
*L.b*_Hemolysin_F	TTGACAAACGGACACAGCCG	
*L.b*_Hemolysin_R	CCTCGCCTCAACACTATTCCATAG	
pLH01_F	CGTTTTGGTCTGCGCGTAAT	Checking the pLH01 (RecE/T) transformation into *L. plantarum*
pLH01_R	CCCCTGACAAGCATCACGAA	
Hly_cleavage_F	CGGTAAACGACGACCGCTAA	Target template PCR for in vitro cleavage assay
Hly_cleavage_R	GTAAGCGCTGTGGGTTCGTA	

## Plasmid transformation by electroporation

To express recombinase RecE/T, plasmid pLH01 (Addgene plasmid # 117261) was transformed by electroporation into *L. plantarum* WCFS1 and *L. plantarum* SPC 72 − 1. For *L. brevis*, plasmid pLH02 (Addgene plasmid #117262) was used to express RecE/T. Electrocompetent cells for plasmid transformation were prepared according to a previous study with several modifications (Huang et al. 2019). An overnight culture was diluted into 100 ml GMRS medium (MRS with 1% glycine) to an optical density at 600 nm wavelength (OD_600_) of 0.1 and incubated at 37 ℃. The cells were harvested by centrifugation at 2504 × g, 4 ℃ when OD_600_ reached 0.4. The cell pellet was washed 3 times with 10 ml of 10mM MgCl₂. The cells were reacted with 800 U/ml of lysozyme at 37 ℃ for 20 min to enhance the transformation efficiency. Then the cells were washed with 1 ml SacGly (10% glycerol with 0.5 M sucrose) or EPS buffer (0.5 M sucrose, 1mM K_2_HPO_4_, 1mM KH_2_PO_4_, 1mM MgCl_2_) and centrifuged at 13,000 rpm for 1 min. The final pellet was resuspended in 50 µl of SacGly or SM buffer (952mM sucrose, 3.5mM MgCl_2_. The competent cells were aliquoted and stored at − 80 ℃. Plasmid DNA 0.2 ~ 1 µg was mixed with 50 µl competent cells and transferred into an ice-chilled 2 mm cuvette. The electroporation was performed with ECM 630 (BTX Harvard apparatus, Holliston, MA, USA) at 2.0 kV, 200 Ω, 25 µF for *L. brevis*, 2.3 kV, 300 Ω, and 25 µF for *L. plantarum*. After electroporation, 900 µl of SMRS broth (MRS with 0.5 M sucrose and 0.1 M MgCl₂) was immediately added to the cuvette and cells were recovered at 37 ℃ for 2–3 h. Finally, the recovery culture was plated on MRS agar containing chloramphenicol (10.0 µg/mL for *L. plantarum* and 6.0 µg/mL for *L. brevis*) and incubated at 37 ℃ for colony selection.

### RNP-mediated CRISPR editing in L. plantarum WCFS1, L. plantarum SPC 72 − 1, and L. brevis SPC-SNU 70 − 2

To optimize the electrocompetent cells for RNP complex electroporation, the protocols of Palomino (Palomino et al. 2010) and Huang (Huang et al. 2019) were combined. *L. plantarum* WCFS1 and *L. plantarum* SPC 72 − 1 with pLH01 and *L. brevis* with pLH02, respectively, were screened using chloramphenicol MRS plates. The transformants carrying pLH01 and pLH02 were cultured in 5 ml of MRS broth with chloramphenicol at 37 ℃. Cells from these cultures were inoculated into MRS medium at an OD_600_ of 0.1. *L. brevis* was grown in MRS broth supplemented with 1% glycine until OD_600_ reached 0.4–0.5. Then the cells were washed with SM buffer 2 times and stored at − 80 ℃. *L. plantarum* cultures were subcultured in MRS medium containing 0.9 M NaCl. Cell growth was stopped at an OD_600_ of 0.6. For all cultures of competent cells, when the OD_600_ reached 0.3, 100 ng/ml of the inducing peptide (MAGNSSNFIHKIKQIFTHR) was added to the medium to induce the recombinase RecE/T. The growth was stopped when OD_600_ reached 0.5–0.6 by cooling cultures on ice for 10 min. The cells were harvested by centrifugation at 4000 rpm, 4 ℃, and washed 3 times with ice-cold ultra-pure water. Electrocompetent cells were aliquoted in 30% PEG 6000 and stored at − 80 ℃. For each electroporation, a frozen aliquot was thawed on ice and used immediately. All RNP electroporations in this study employed frozen competent cells; we did not perform a head-to-head comparison with freshly prepared cells.

For the RNP complex, Alt-R™ S.p. Cas9 Nuclease V3 and Alt-R™ CRISPR-Cas9 sgRNAs (Integrated DNA Technologies) were used in this experiment, with the sgRNA sequences are listed in Table 1. The pre-assembled RNP complex was electroporated into RecE/T-expressing strains. Specifically, 80 µg of Cas9 nuclease, a total of 60 µg of two sgRNAs, and 80–100 µg (2–3 µl) of repair template were mixed with 50 µl of competent cells (approximately 1.2 × 10⁶ cells per 50 µl for *L. plantarum* and 2.3 × 10⁴ cells per 50 µl for *L. brevis*) and incubated on ice for 20 min. The mixture was transferred to 1 mm ice-cold cuvettes (BTX, USA) and electroporation was conducted by ECM 630 (BTX, USA) under the following conditions: 1250 V, 200 Ω, and 25 µF. To enhance delivery efficiency, electroporation parameters (voltage, resistance, pulse number, and cuvette gap) were systematically evaluated. In addition, dual sgRNAs were designed in a PAM-out orientation, and the ratio of sgRNA to Cas9 protein was varied to identify the most effective RNP composition. After electroporation, 900 µl of recovery medium (MRS supplemented with 0.5 M sucrose and 0.1 M MgCl_2_) was added to the cuvette and recovered for 3 h at 37 ℃. The cells were then pelleted, washed three times with PBS, and plated on MRS agar containing chloramphenicol (10.0 µg/mL for *L. plantarum* and 6.0 µg/mL for *L. brevis*) to select RecE/T-expressing transformants.

## Detection of mutations

Colony PCR was used to select mutants with a 50 bp deletion of *hlyIII* and the insertion of a stop codon (TAA). The mutant colony had a 47 bp smaller DNA fragment than the wild-type *hlyIII*. Both methods were used to extract the colony DNA. First, the colony was suspended in 20 µl of 10mM NaOH and incubated for 15 min at 98 ℃. To remove cell debris, the colony suspension was centrifuged for 15 min at 13,000 rpm. The 2 µl of supernatant was used for colony PCR. The other DNA extraction protocol for Gram-positive bacteria was described by (Chen and Yuan 2023). The single colony was picked and transferred to 20 µl of phosphate-buffed saline (PBS). Sonication was performed for 5 min using a Shin Han cleaner (Shin Han Ultrasonic Waves, Korea). The sonicated colonies were centrifuged at 10,000 g for 1 min. A volume of 2–3 µl of the supernatant was added to the reaction. The mixture was combined with the AccuPower PCR Master Mix (Boineer, Daejeon, Korea). The colony PCR assays were implemented by SlimpliAmp Thermal Cycler (Thermo Fisher Scientific, Waltham, MA, USA) using a pair of primers flanking outside the target site of the *hlyIII* gene on the chromosome. The primers used for colony PCR of *hlyIII* are listed in Table 1. The PCR products were loaded onto a 2% agarose gel (Dyne Bio, Sungnam, Korea) and electrophoresis was conducted at 100 V, 30 min, utilizing Mupid-exU (Takara Bio Inc., Kusatsu, Shiga, Japan). A 25/100 bp DNA ladder (Bioneer, Daejeon, Korea) was loaded in the first lane to compare the DNA sizes. In the second lane, H_2_O was loaded as a negative control, and the genomic DNA of the wild type was loaded in the third lane as a positive control.

### Statistical analysis

Statistical analysis was carried out using Microsoft Excel. Statistical significance was determined using paired-sample t-tests. A p-value of less than 0.05 indicated a significant difference between data sets. Data were collected from three independent replicates, and the mean values were used for the analysis (Xu et al. 2014).

## Hemolytic assay

The hemolytic activities were determined on 5% sheep blood agar plates and through a quantitative hemolytic assay using human single-donor whole blood (Innovative Research, USA). Wild-type strains and ∆*hlyIII* mutant colonies were picked and cultured in 5 ml of MRS medium. The cell culture was centrifuged to collect the cell pellets, which were resuspended in PBS at an OD_600_. Suspended bacterial cells were plated on a 5% sheep blood agar plate (Bandio, Pocheon, Korea), which was then incubated at 37 ℃. The hemolytic zones on sheep blood agar generated by the mutant and wild-type strains were compared. *HlyIII* inactivated mutants with reduced hemolytic activity were isolated. *Streptococcus mutans*, *Staphylococcus aureus*, and *Enterococcus faecalis* were cultivated as the α-, β-, and γ- hemolysis control, respectively. Streaking of the control strains was performed under the same conditions. The plates were observed under a light source to confirm the hemolytic effect on the blood agar plate.

The quantitative hemolytic activity was tested according to Ridder (Ridder et al. 2021) and a biomaterial hemolytic assay kit (Haemoscan, Groningen, Netherlands). Human single-donor whole blood (Innovative Research, Inc., MI, USA) was used to prepare the erythrocytes. Blood samples (1 ml) were transferred to a 15 ml conical tube and gently mixed with 12 ml cold 1× PBS. Then the erythrocytes were centrifuged at 1450 × g for 10 min at 4 ℃. The supernatant was removed, and the cells were washed three times with 1× PBS. The final concentration was 4% (v/v) after resuspension in 1× PBS. In addition, human blood for the biomaterial hemolytic assay kit was prepared according to the manufacturer’s protocol. Mutants were cultured in MRS broth at 37 ℃, 18 h, and the cell population was adjusted to 3.0 × 10^7^ cfu/ml. 0.2 ml of erythrocytes and the same volume of cell culture were mixed and incubated at 37 ℃, 24 h. 1% Triton X-100 and 1× PBS were used as the positive and negative controls, respectively. After 24 h of incubation, the test compound was centrifuged at 1000 × g for 10 min. Then, 20 µl of the supernatant from the sample or control was transferred with 180 µl of PBS to a transparent flat-bottom 96-well plate, and absorption at 450, 380, and 415 nm was measured in a plate reader. The final concentration of free hemoglobin in the supernatant was determined using the Harboe method. Free hemoglobin levels were calculated using the equation, fHb [µM] = 0.155 × 83.6 (2 × A_415nm_ – A_380nm_ – A_450nm_) (Chung et al. 2020; Marie et al. 2024).

## Results

### Establishment of RNP-mediated CRISPR-Cas9 editing in L. plantarum WCFS1

**Fig. 1:**
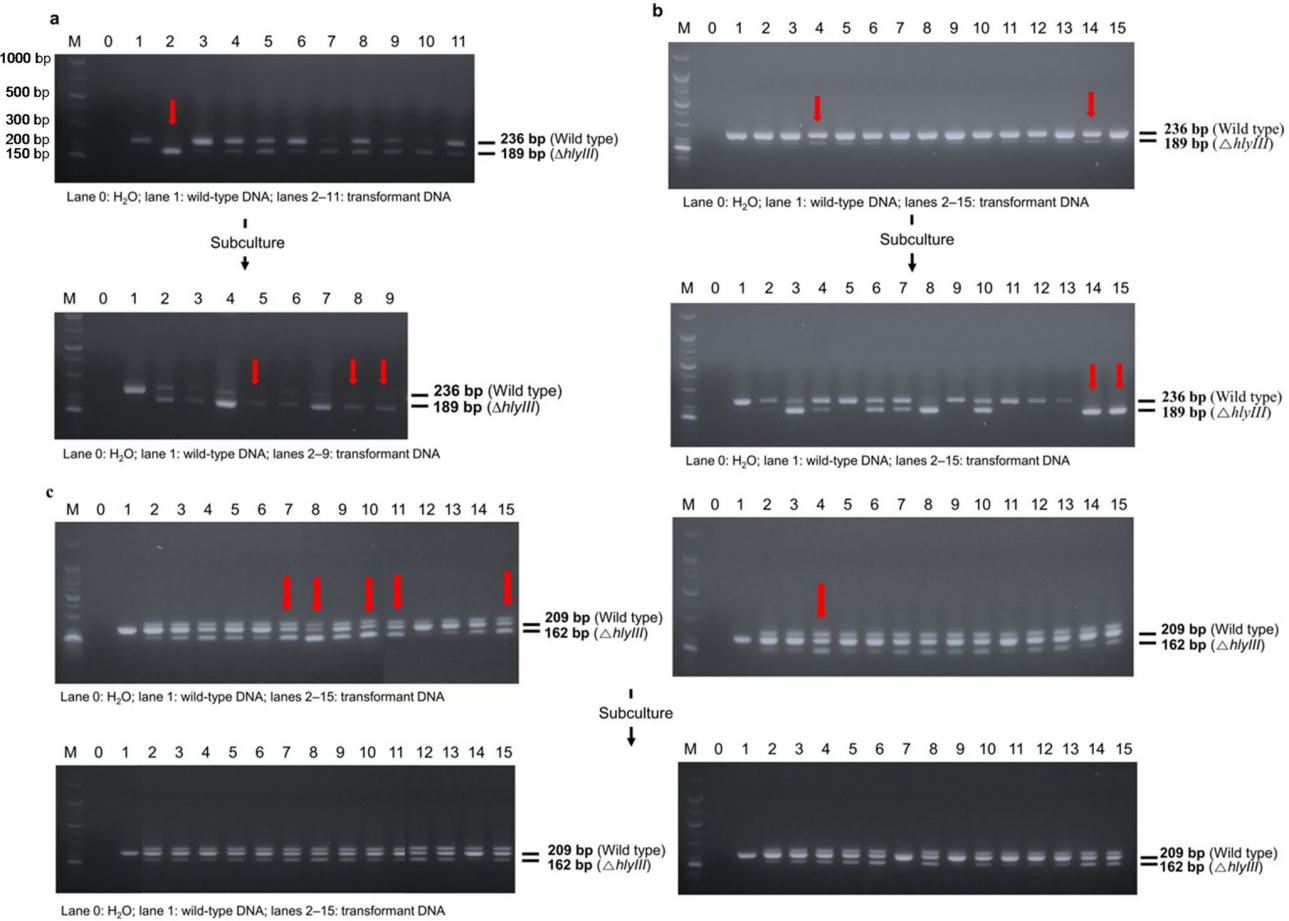
Quantitative hemolysis of wild-type strains and *∆hlyIII* mutants. **(a)** *L. plantarum* WCFS1, **(b) ***L. plantarum* SPC 72 − 1, and **(c)** *L. brevis* SPC-SNU 70 − 2. The figure shows the concentration of free hemoglobin after incubation of cultures with human erythrocytes at 37 ℃ for 24 h. Values represent the mean ± SD of three independent experiments. The average values of 4–5 independent mutants are shown. Data below 0.2 and 0.05 are omitted by a break line. Significant differences are indicated by asterisks (*)

**Fig. 2:**
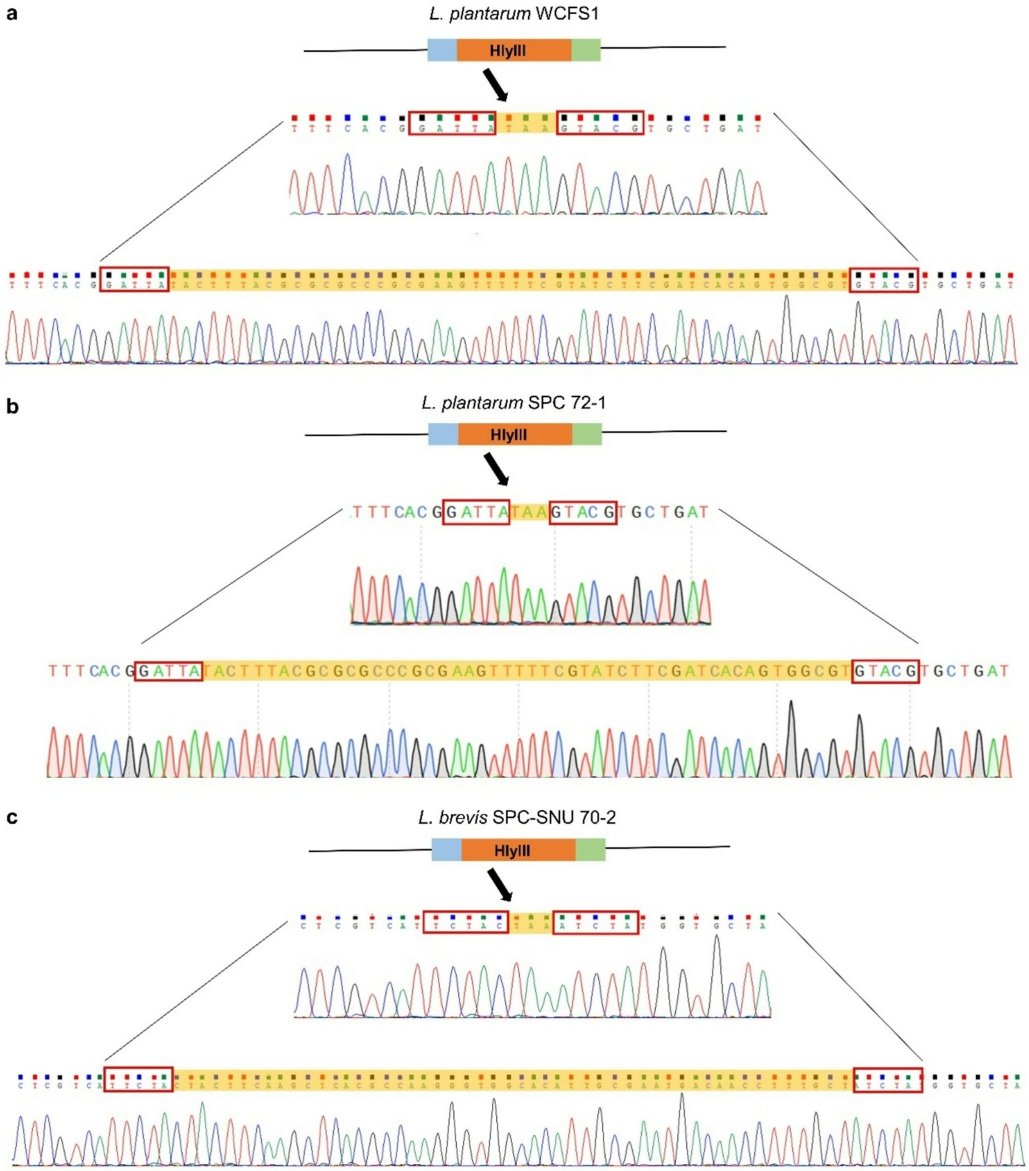
Result of PCR analysis of RNP transformants targeting *hlyIII* in **(a)** *L. plantarum* WCFS1, **(b)** *L. plantarum* SPC 72 − 1, and **(c)** *L. brevis* SPC-SNU 70 − 2. M: 25/100 bp DNA ladder Lane 0: H_2_O (negative control) Lane 1: Genomic DNA of wild type Lane 2–15: transformants DNA. The DNA fragments of the wild type and mutants with inactivated *hlyIII* are shown on the gel. The red arrows indicate the subcultured colonies for the next generation. The colonies were analyzed by colony PCR

Construction of an *hlyIII* inactivated mutant of *L. plantarum* WCFS1 requires a Cas9 nuclease, sgRNA targeting *hlyIII*, and a repair template for transformation into *L. plantarum* WCFS1 to induce homologous recombination. This experiment used ribonucleoprotein (RNP) for CRISPR-Cas9 editing instead of the plasmid-carrying cas9 protein, which allows genome editing without antibiotic resistance markers. However, its effectiveness largely depends on the transformation capability of the bacterial strain and the recombination machinery, which refers to cellular components such as RecA and other recombinases that mediate homologous recombination and facilitate the integration of donor DNA. Many lactic acid bacteria exhibit limited native recombination activity, making genome editing challenging without the assistance of exogenous recombinases, such as RecE and RecT. Moreover, the thick, highly cross-linked peptidoglycan layer of Gram-positive bacteria presents an additional barrier to efficient electroporation, unlike that of Gram-negative bacteria, which have relatively thin envelope structures. This structural challenge can significantly hinder the delivery of genome-editing complexes into cells (Arroyo-Olarte et al. 2021). Competent cells grown in a high-salt medium were used to improve electroporation efficiency, as such conditions have been shown to enhance membrane permeability and facilitate DNA uptake in recalcitrant strains. According to a previous study, *Lactobacillus casei* BL23 grown in NaCl-containing medium showed a decrease in peptidoglycan cross-linking involving penicillin-binding proteins (PBP) in cells experiencing osmotic stress (Piuri et al. 2005). Furthermore, to improve the efficiency of homologous recombination, the plasmid expressing RecE/T was transformed into wild-type strains, according to a previous study (Huang et al. 2019). The electroporation of RNP targeting *hlyIII* with repair DNA was conducted to develop the ∆*hlyIII* mutants. For the RNP complex, Cas9 nuclease and sgRNAs designed to target and cleavage *hlyIII*, were used. The efficiency of sgRNA in cleaving the targeted sequence was tested using an in vitro cleavage assay with Hly_cleavage_F and Hly_cleavage_R (Table 1). Among the sgRNA candidates, L. p _Hem_sgRNA2 and 17 showed the highest cleavage efficiencies and were selected to form the RNP complex. The electroporated RNP complex cleaves the target DNA, and the repair DNA induces HDR to generate a 50 bp target sequence deletion and stop codon (TAA) insertion between the homologous arms. The predicted transformants were analyzed by colony PCR to verify whether the desired mutations had occurred. As shown in the agarose gel, both *hlyIII*-inactivated mutants (single band) and *hlyIII*-inactivated mutants (mixed band) were observed (Fig. 1a). The mutants were subcultured and streaked onto an MRS plate. The transformed cells were subsequently subcultured and plated on MRS agar supplemented with chloramphenicol to facilitate the selection and isolation of wild-type colonies and genetically homogeneous mutant strains. After the first subculture, 96.4% of colonies contained *hlyIII*‐inactivated mutants, and 3 had 189 bp of a single band. However, when the mutants that presented a single band reached the second subculture, most showed mixed DNA bands (236 and 189 bp) on the agarose gel (Fig. 1a). In addition, sequencing revealed that the target sequences were edited as repair templates with a 50 bp deletion and stop codon insertion (Fig. 2a). When examining the colony PCR results of the *∆hlyIII* mutant (Fig. 1a), mixed-genotype colonies displaying both wild-type and mutant bands were observed. Although *Lactobacillus* species are haploid organisms, these colonies have been observed after CRISPR-Cas9 RNP-based editing. This likely resulted from incomplete allelic segregation or mosaic editing during early cell division after transformation. In these colonies, most cells retained the wild-type allele, whereas some carried the intended mutation. To obtain genetically homogeneous strains, mixed colonies were subcultured, allowing the isolation of pure mutant populations.

### Establishment of RNP meditated CRISPR-Cas9 editing in L. plantarum SPC 72 − 1

The *hlyIII* sequences of *L. plantarum* SPC 72 − 1 and *L. plantarum* WCFS1 were compared to determine whether RNP-mediated CRISPR-Cas9 can be applied to *L. plantarum* SPC 72 − 1. As a result of the alignment of *hlyIII* genes, the sequences were 100% identical. Therefore, the same sgRNA and repair template were used to inactivate *hlyIII* in *L. plantarum* SPC 72 − 1. To inactivate the *hlyIII* gene in *L. plantarum* SPC 72 − 1, we used the same protocol as that used for *L. plantarum* WCFS1. This included preparing electrocompetent cells using a high NaCl concentration, transforming cells with a helper plasmid, and performing RNP electroporation targeting the *hlyIII* gene. The colonies of wild type and ∆*hlyIII* inactivated mutants were amplified with 236 bp and 189 bp, respectively, by a pair of primers flanking near the target site. The 71.4% of mutants represented in the first generation showed a mixed band, which means the coexistence of wild type and ∆*hlyIII* inactivated mutants. No single band was observed on the gel. The mixed mutants were then subcultured on MRS plates with chloramphenicol to separate the wild type and pure mutants. After the first subculture, two single-band mutants with 189 bp fragments were detected, with 85% of colonies containing *hlyIII*-inactivated mutants (Fig. 1b). Furthermore, sequencing confirmed deletion and insertion of the repair template into *L. plantarum* SPC 72 − 1 (Fig. 2b). Mixed-genotype colonies were also observed for *L. plantarum* SPC 72 − 1. However, unlike *L. plantarum* WCFS1’s PCR results, all mutants showed mixed bands on the gel in the first analysis and a more apparent single band after subculture. This result indicated that the efficiency of genome editing using the RNP complex depends on the strain.

### Establishment of RNP-mediated CRISPR-Cas9 editing in L. brevis SPC-SNU 70 − 2

An advantage of using CRISPR-Cas9 with RNP is that it expedites the process of strain preparation and gene editing by enabling application to different bacterial strains with specifically designed sgRNAs and repairing DNA containing homologous arms without the need for constructing plasmids. The versatility of the *L. brevis* SPC-SNU 70 − 2 was selected for editing. The same gene, *hlyIII*, was also used to establish the RNP-mediated method. Electroporation conditions and competent cells for *L. plantarum* were optimized for *L. brevis* SPC-SNU 70 − 2 with a few modifications. Instead of growing cells in a high-salt medium, various cell wall-weakening agents (glycine, lysozyme, and penicillin G) adapted to *L. plantarum* WCFS1 and *L. plantarum* SPC 72 − 1 were utilized to improve the transformation efficiency of the RNP complex. Transformants were prepared by electroporation of treated competent cells, which were then analyzed by colony PCR. The PCR products of *L. brevis* showed unexpected results, with three bands: wild-type (209 bp), mutant (162 bp), and an unknown upper band (Fig. 1c). Most colonies showed three bands on the agarose gel, indicating that the target gene was cleaved and repaired by HDR. After subculturing, the mutant band was maintained with unknown bands, which were assumed to be a heteroduplex form of the wild-type and mutant, a phenomenon also observed by Qi and co-workers (Qi et al. 2016). Sequencing analysis of the mutant band showed a 50 bp deletion and stop codon insertion in the desired sequences (Fig. 2c). PCR analysis of *L. brevis* SPC-SNU 70 − 2 revealed three bands on the agarose gel, of which only the upper band was extracted using the gel extraction method. Electrophoretic analysis of the extracted DNA revealed two distinct bands corresponding to the wild-type and mutant alleles, despite careful excision of the upper band during gel extraction. The two bands were well separated on the gel with sufficient spacing to minimize the risk of cross-contamination during the extraction process. This result is similar to that of Qi’s study, which showed noisy signals in the sequencing chromatograms of the upper band, even when a single colony was picked. Through systematic optimization of electroporation parameters, sgRNA design, and RNP composition, we achieved a significant improvement in editing efficiency, resulting in a higher proportion of desired mutants.

### Comparison of hemolysis of hlyIII inactivated mutant with wild type

**Fig. 3:**
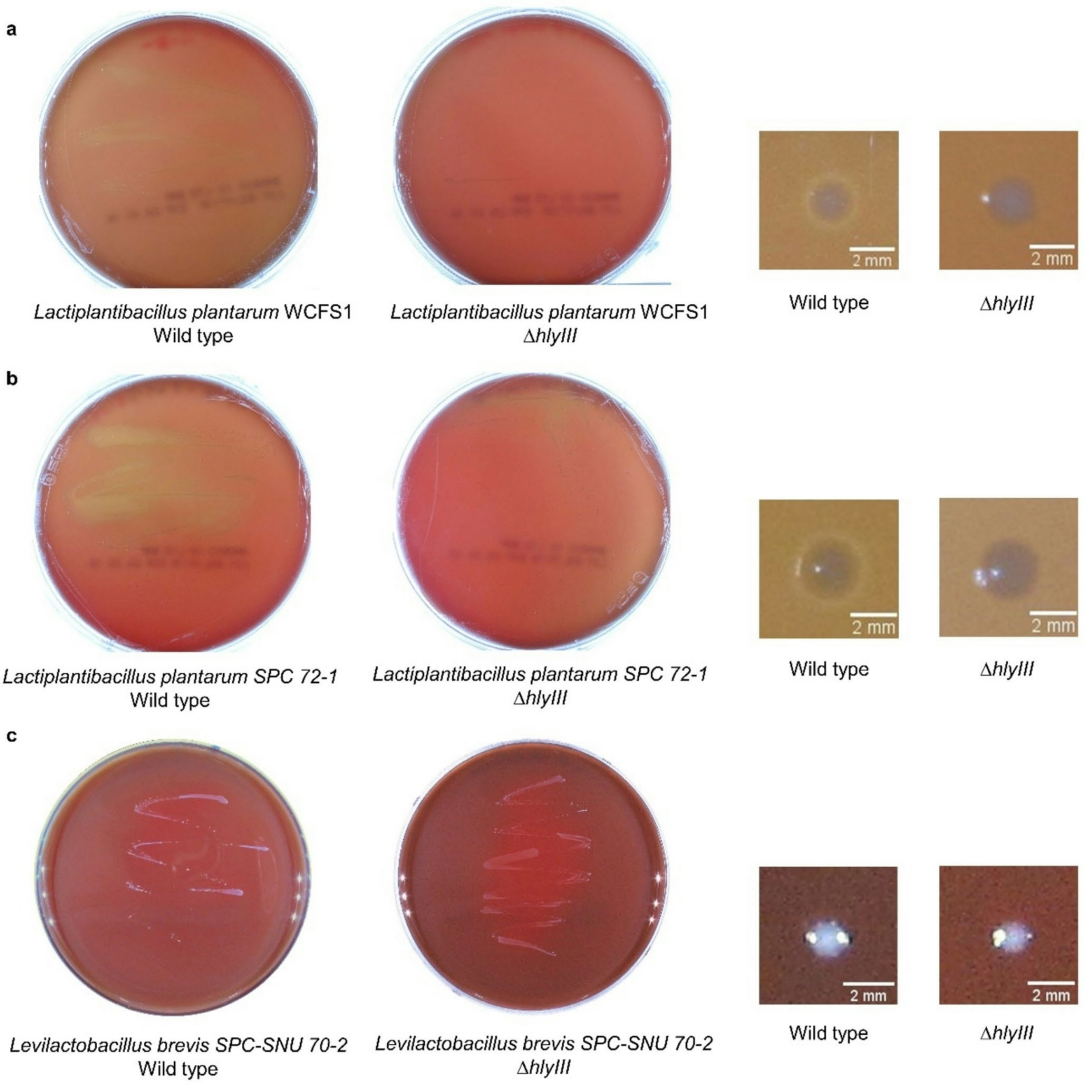
Sequencing data of *∆hlyIII* mutants. **(a)** *L. plantarum* WCFS1, **(b)** *L. plantarum* SPC 72 − 1, and **(c)** *L. brevis* SPC-SNU 70 − 2. Chromatograms of the wild-type strains and corresponding *hlyIII*-inactivated mutants are shown. Sequencing results were analyzed using Chromas (Technelysium, South Brisbane, Australia) and SnapGene (GSL Biotech LLC, Boston, MA, USA)

The difference in hemolysis between the wild-type strain and the *hlyIII*-inactivated mutants of *L. plantarum* WCFS1 was tested in two ways. First, a hemolysis test using 5% sheep blood agars was conducted, which is commonly used to phenotype bacterial hemolysis. Although the differences between colonies were clearly observable, numerically quantifying these differences was challenging. Therefore, a quantitative hemolytic activity assay using human erythrocytes was conducted to precisely measure hemolytic differences. On a 5% sheep blood agar plate, *L. plantarum* WCFS1 and *L. plantarum* SPC 72 − 1 exhibited α-hemolytic activity, producing greenish discoloration around the colonies and in the surrounding medium. The phenotype appeared to be a reduction of red blood cell hemoglobin to methemoglobin (Buxton 2005). In contrast, the *ΔhlyIII* mutants showed minimal greenish or brownish coloration, and the halo areas around their colonies after 24 h of incubation were smaller and less distinct than those of the wild type (Fig. 3a, b). The wild-type strain of *L. brevis* SPC-SNU 70 − 2 displayed gamma hemolysis, characterized by the absence of hemolysis and no visible color change around the colonies (Fig. 3c). *Lactobacillus* spp. typically exhibit alpha-hemolysis or gamma-hemolysis when grown on blood agar (Goldstein et al. 2015), with strain-dependent variations in hemolytic patterns (Shucen et al. 2023), which aligns with our observations.

**Fig. 4:**
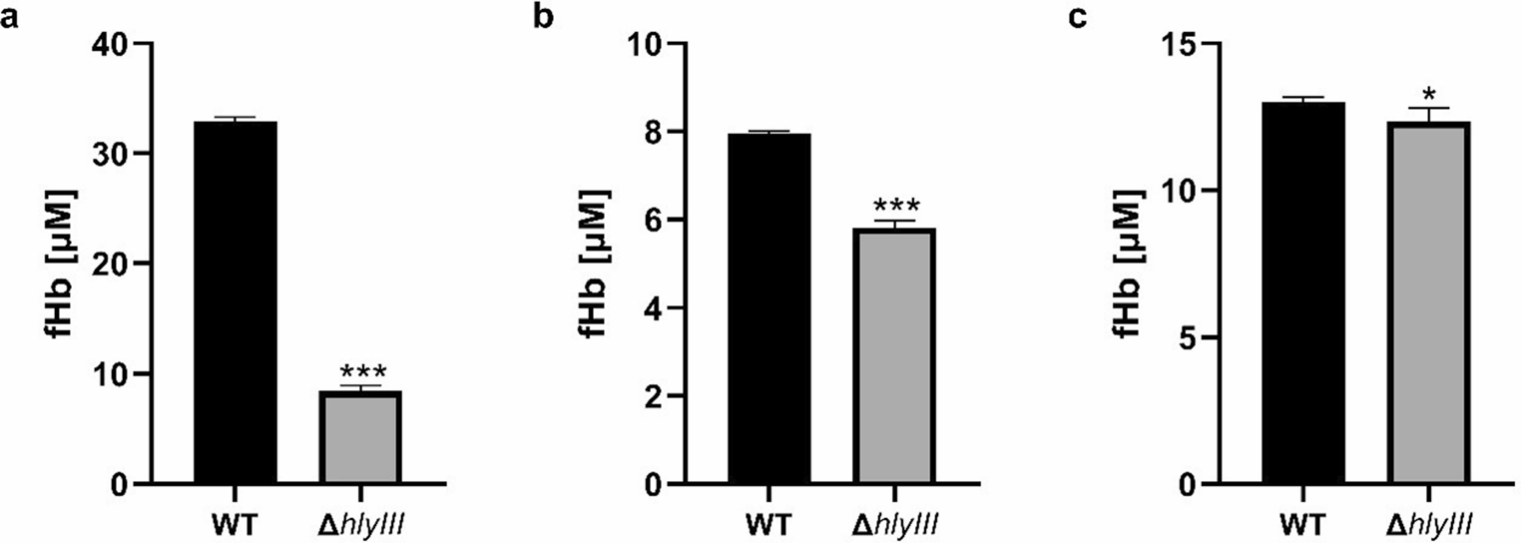
Comparison of hemolysis phenotypes between wild-type strains and *∆hlyIII* mutants on 5% sheep blood agar. **(a)** *L. plantarum* WCFS1, **(b)** *L. plantarum* SPC 72 − 1, and **(c)** *L. brevis* SPC-SNU 70 − 2. Hemolysis phenotypes and hemolytic zones were observed on blood agar plates after 48 h of incubation

A quantitative hemolytic activity assay was performed using human blood after 24 h of incubation, similar to the probiotic intake circumstances in humans. This method is based on the release of hemoglobin, which can be spectrophotometrically measured. As more hemolysis occurred, the free hemoglobin concentration in the supernatant increased. A standard curve of hemoglobin was obtained using various final concentrations of the hemoglobin solution (0, 0.15, 0.31, 0.63, 1.25, 2.5, 5, and 10 mg/mL). Hemoglobin concentration was determined based on the absorbance of Hb in the supernatant. For the test, *L. plantarum* WCFS1 ∆*hlyIII* mutants M3-1, M3-2, M3-3, M24-1, M24-2, *L. plantarum* SPC 72 − 1 ∆*hlyIII* mutants M7-1, M7-2, M8-1, M8-2, and *L. brevis* SPC-SNU 70 − 2 ∆*hlyIII* mutants M34-23, M34-24, M34-28, M51-49 were picked for hemolysis test. Erythrocyte suspension was incubated with an equal volume of ∆*hlyIII* mutant culture. The results showed that the hemoglobin concentration of all colonies tested was lower than that of the wild type for both strains. Compared to the wild type, the *L. plantarum* WCFS1 and *L. plantarum* SPC 72 − 1 *∆hlyIII* mutants showed an average decrease in hemoglobin of 27.0% and 74.3%, respectively (Fig. 4a and b). *L. brevis* SPC-SNU 70 − 2 showed a 5.0% decrease in hemoglobin concentration compared to the wild type (Fig. 4c). This result was significantly lower than the reduction in the levels of the two *L. plantarum* strains. This showed a trend similar to that of the experiment conducted on the blood agar plate. In conclusion, *ΔhlyIII* mutants exhibited a statistically significant reduction in hemolytic activity against human erythrocytes (*p* < 0.05). Since α-hemolysis is thought to result from the conversion of hemoglobin to methemoglobin by hydrogen peroxide (H₂O₂) produced by bacteria (Lad et al. 2022), our findings may suggest a decrease in H₂O₂ production in *L. plantarum* WCFS1 following *hlyIII* inactivation. However, further experiments are needed to clarify the mechanistic link between *hlyIII* and H₂O₂-mediated hemolysis.

## Discussion

We optimized RNP-mediated genome editing in *L. plantarum* WCFS1 and *L. plantarum* SPC 72 − 1, successfully generating *ΔhlyIII* mutants with editing efficiencies of 96.4% and 85%, respectively. We found that efficient recombinase activity and homologous arms longer than 500 bp are both essential for proper homologous-directed repair (HDR) in lactic acid bacteria. In this experiment, the recombinase protein RecE/T was transformed into target strains to enhance the recombineering efficiency. Pijkeren and Britton (van Pijkeren and Britton 2012) reported recT-assisted ssDNA mutagenesis in *L. reuteri* and other LABs, including *L. lactics*, *L. gasseri*, and *L. plantarum*. Furthermore, RecE/T-mediated HDR using dsDNA was successful in *L. plantarum* and *L. brevis* (Huang et al. 2019). We also used RecE/T-mediated HDR to demonstrate its essential role in efficient genome editing. Nevertheless, this study provides a methodological advance by establishing an efficient transformation method using a protein and RNA complex (RNP). This system eliminates the need for plasmid construction and cloning, which were labor-intensive in previous approaches. Additionally, it avoids potential plasmid stability issues that could affect the consistent supply of the Cas protein and sgRNA. Moreover, the construction of various plasmids introduces several constraints. Therefore, the development of an RNP-based method provides a meaningful alternative to overcome these limitations and ensure a more efficient and stable genome editing system.

While we successfully achieved a 50 bp deletion and stop codon insertion in the hemolysin gene, we consistently observed the presence of mixed-genotype colonies across all target strains (*L. plantarum* SPC 72 − 1, *L. plantarum* WCFS1, and *L. brevis* SPC-SNU 70 − 2). These colonies displayed both wild-type and mutant bands. This phenomenon, also observed in other CRISPR-Cas9 studies (Huang et al. 2019), likely stems from factors such as insufficient sgRNA transcription, inefficient chromosomal breaks, or the rapid growth of unedited wild-type cells in the absence of selection pressure. The tendency of *L. brevis* SPC-SNU 70 − 2 to form cell aggregates and engage in genome exchange, possibly mediated by EPS production, might further contribute to the persistence of mixed genotypes through incomplete homologous recombination or heterozygous mutations resulting from single-crossover events. In line with this, Chen’s study (Chen et al. 2021) showed that heterozygous mutants generated by in vivo single-cross recombination events maintained their heterozygous genotypes even after repeated subculturing under selection pressure, paralleling our observations in *L. plantarum* SPC 72 − 1, *L. plantarum* WCFS1, and *L. brevis* SPC-SNU 70 − 2. Furthermore, the unexpected heteroduplex bands observed in *L. brevis* SPC-SNU 70 − 2 also strongly suggest mixed-genotype formation (Qi et al. 2016). While pure mutants can theoretically be isolated through subculturing (Song et al. 2017), achieving complete genetic homogeneity proved to be very challenging in our experiments. This observation highlights a limitation in achieving complete genetic homogeneity. The presence of mixed genotypes suggests a potential for reversibility, reminiscent of non-heritable gene inactivation methods like RNA interference (RNAi) or CRISPR interference (CRISPRi). However, unlike these methods where a selectable marker on a plasmid often helps maintain the phenotype under selective pressure, our RNP-based approach lacks such a selection pressure, making the sustained maintenance of the desired phenotype more challenging. Despite these challenges, this novel method offers a versatile platform for inactivating potential virulence genes and readily editing other genes by synthesizing specific gRNA and templates. Further improvements in recombination efficiency and strategies to minimize cell aggregation are crucial for achieving more consistent genome editing and isolating pure mutants more effectively.

The *ΔhlyIII* mutants exhibited significantly reduced hemolytic activity compared to wild-type strains, raising the possibility that deletion of hemolysin-related genes may influence hemolytic phenotypes in certain strains. Similar findings have been observed in other pathogens. For instance, a deletion mutant of hemolysin-III-related protein (Hhly3) in *Streptococcus suis* serotype 2 demonstrated a significant reduction in hemolytic activity compared to the wild-type (Zheng et al. 2013), while in *Vibrio parahaemolyticus*, the deletion of *hlyA* and *hlyIII* reduced hemolytic activity by 31.4% and 24.9%, respectively (Jinyuan et al. 2024). Although previous studies have implicated *hlyIII* in hemolytic activity in pathogens such as *Bacillus cereus* (Gleb and Nikolai 1996) and *Vibrio vulnificus* (Chen et al. 2004), its role in lactic acid bacteria remains unclear. Existing data suggest that LAB strains typically test negative for hemolytic activity, or exhibit only α-hemolysis, which is not associated with pathogenicity and is distinct from β-hemolysis that causes complete red blood cell lysis (Oh et al. 2022; Nawaz et al. 2024; Kiani et al. 2025; Sitdhipol et al. 2025). Thus, while our findings suggest that *hlyIII* deletion may affect hemolytic phenotypes in certain LAB strains, these results remain preliminary and require further validation through carefully controlled assays. Notably, the presence or annotation of *hly*-like genes in LAB genomes does not necessarily imply functional expression or correlate with measurable hemolysis, and their physiological roles in LAB remain unresolved.

In summary, this study introduces a versatile RNP-mediated genome editing approach applicable to various LAB strains, significantly reducing editing time and costs, and eliminating residual foreign genes compared to traditional plasmid-based methods. Despite these advantages, the current reliance on the RecE/T recombinase necessitates the use of plasmids, highlighting the need to develop alternative strategies to achieve a fully protein- and RNA-based RNP system. Accordingly, the workflow is not yet fully plasmid‑free. We did not evaluate co‑delivery of purified RecE/T proteins with the RNP complex in this study. Future work will test plasmid‑independent recombinase delivery (e.g., purified protein or mRNA) or transient chromosomal expression with counter‑selection to achieve a fully plasmid‑ and antibiotic‑free workflow. Additionally, all RNP electroporations in this study used frozen electrocompetent cells stored at − 80 ℃; we did not benchmark against freshly prepared cells, which may influence absolute transformation efficiency. Furthermore, challenges remain in achieving complete genetic homogeneity, as mixed-genotype colonies were frequently observed across target strains. The absence of antibiotic-based selection pressure in the RNP-based system may contribute to this limitation. Addressing such issues—through enhanced recombination strategies and minimization of cell aggregation—will be essential for reliable mutant isolation and stable phenotype expression. Through systematic optimization of electroporation parameters, sgRNA design, and RNP composition, we achieved a significant improvement in editing efficiency, resulting in a higher proportion of desired mutants. Nevertheless, by enabling the precise editing of chromosomal genes in LAB, including those annotated as potential safety-related elements, this platform may expand the potential of probiotic engineering and enable the development of LAB-based biotherapeutics with improved safety, tailored functionalities, and therapeutic efficacy. Ultimately, engineered probiotics hold promise for addressing global health challenges, including their use as delivery platforms for vaccines and therapeutics targeting infectious diseases and cancers (Mugwanda et al. 2023).

## Data Availability

No datasets were generated or analysed during the current study.
